# Application of the Finite Element Method to the Incremental Forming of Polymer Sheets: The Thermomechanical Coupled Model and Experimental Validations

**DOI:** 10.3390/polym12081715

**Published:** 2020-07-30

**Authors:** A. García-Collado, Gustavo Medina-Sanchez, Munish Kumar Gupta, R. Dorado-Vicente

**Affiliations:** 1 Department of Mechanical and Mining Engineering, University of Jaén, EPS de Jaén, Campus Las Lagunillas, 23071 Jaén, Spain; gmedina@ujaen.es (G.M.-S.); rdorado@ujaen.es (R.D.-V.); 2 Key Laboratory of High Efficiency and Clean Mechanical Manufacture, Ministry of Education, School of Mechanical Engineering, Shandong University, Jinan 250061, China; munishguptanit@gmail.com

**Keywords:** parallel rheological framework, Finite Element Method (FEM), incremental forming, thermoplastic

## Abstract

Single Point Incremental Forming (SPIF) is an innovative die-less low-cost forming method. Until now, there have not been viable numerical solutions regarding computational time and accuracy for the incremental forming of polymers. Unlike other numerical approaches, this novel work describes a coupled thermomechanical finite element model that simulates the SPIF of polymer sheets, where a simple elastoplastic constitutive equation rules the mechanical behavior. The resulting simulation attains a commitment between time and accuracy in the prediction of forming forces, generated and transmitted heat, as well as final part dimensions. An experimental test with default process parameters was used to determine an adequate numerical configuration (element type, mesh resolution, and material model). Finally, compared to a set of experimental tests with different thermoplastics, the proposed model, which does not consider complex rheological material models, shows a good agreement with an approximation error of less than 11% in the vertical forming force prediction.

## 1. Introduction

**Figure 1:**
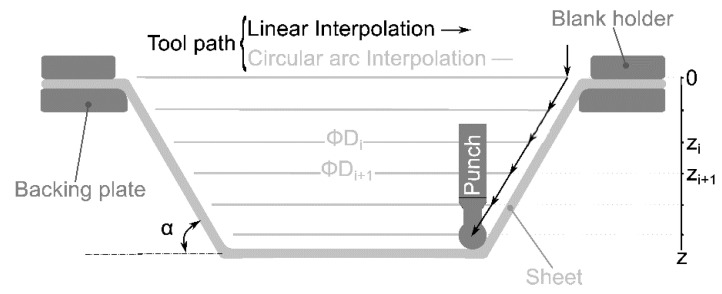
Single point incremental forming scheme and tool path implemented for the numerical and experimental tests accomplished in this work.

Single point incremental forming (SPIF) is the most straightforward implementation of the original idea of Leszak [1] for incremental sheet forming (ISF). In a SPIF operation, the movement of a small hemispheric tool, usually Computer Numerical Control (CNC) controlled, deforms a sheet of material, fixed to a rigid frame by a holder, producing a 3D shape (Figure 1).

High formability and the low-cost die-less setup required are the SPIF advantages appreciated by the automotive, aerospace, and medical sectors. On the other hand, the SPIF process is slower than conventional forming processes and faced problems of dimensional accuracy, thinning, and surface finishing. It is essential to overcome the drawbacks mentioned above to take advantage of SPIF formability and flexibility. The study of the deformation mechanism and material behavior can help to improve the process.

During the last two decades, experimental and numerical simulations were conducted to know the deformation mechanism and material behavior in SPIF as a previous step to improve the process. Most studies are focused on the relation among SPIF parameters and formability, resulting in forces and final shape, which is key to understand the process.

Different review works were reported to sort the extensive knowledge produced about the incremental forming processes. Gatea et al. [2] gathered information from experimental and numerical studies about the observed influence in metal sheets formability, failure, spring-back, and surface quality of the main incremental forming parameters.

The work of Li et al. [3], also for metal sheets, added a discussion of forming forces prediction to the review of studies about the deformation mechanism, formability, and geometry. Regarding forming force prediction, these authors claim that the numerical approaches are challenging to implement due to its computational time. On the other hand, analytical solutions are faster but not accurate.

Behera et al. [4] mentioned the extension, in the last decade, of SPIF from metals to other materials such as polymers, composite panels, and shape memory alloys, and included studies about the incremental forming of polymer sheets in their revision of the SPIF progress. The extension to different materials was also noted by McAnulty et al. [5], whose work about formability considers contributions about SPIF of metal and polymer sheets.

Regarding polymer sheets, Franzen et al. [6] proved that Polyvinyl Chloride (PVC) could be incrementally formed at room temperature. Le et al. [7] tested and checked the influence of SPIF process parameters on the polypropylene formability. Later, Martins et al. [8] identified the critical process parameters and material properties in the incremental forming of five polymers: Polyethylene (PE), Polyoxymethylene (POM), Polyvinyl Chloride (PVC), Polyamide (PA) and Polycarbonate (PC). Marques et al. [9] used Fracture Forming Limit Diagrams (FFLD) to characterize polymers since these materials show an absence of necking before failure. Besides, they studied the optimal setup through different experiments and assessed the results qualitatively using a theoretical membrane analysis and pressure-sensitive yield surfaces [10].

More recent studies noted that an increment of the spindle speed improves the formability of polymers because the punch-sheet friction increases the temperature [11]. Using a semi-analytical model, Medina-Sánchez et al. [12] proposed a semi-analytical model to predict the incremental forming force for polymers and compare the results with experimental measurements and numerical simulations. The last research works study novel polymeric materials and applications, for example, the ultra-high molecular weight polyethylene material reinforced with nanoparticles of TiO_2_ developed by Ortiz-Hernández et al. [13], or the composite based on PA matrix and clay filler studied by Borić et al. [14].

Regarding numerical studies about polymers, the material model plays an essential role due to stress relaxation after yielding, viscoplasticity, and temperature-dependent effects. An adequate mechanical model is enough for metals, but not for polymers. A thermal approach is key since the temperature increment due to friction effect between the punch and blank added to generate heat by the plastic deformation, reduces the maximum reaction force, increases the formability, and modifies the material behavior [11].

Advanced thermomechanical constitutive models of parallel-connected networks capture the above-mentioned behavior for polymers [15]. Still, these models require sizable computational time and are not indicated to simulate SPIF processes. Recently, Yonan et al. [16] proposed a viscoplastic model for thermoplastic materials, which uses an implicit scheme to simulate incremental forming at room temperature, and low tool velocity. Nevertheless, this approach is limited to large deformations and a small number of increments at room temperature. On the other hand, Bergstrom [17] showed that if the deformation does not exceed the limit where the viscoplasticity is more accurate than plasticity, then some thermoplastics can be modeled with classical J_2_-plasticity material equation with a 5–10% error. 

This paper describes a thermo-mechanical model for the SPIF of thermoplastic sheets. Although this model focuses on forming forces prediction, the solution also provides temperature distribution, final shape, and thickness. Moreover, it takes into account the temperature effects due to friction using temperature-dependent material with simple hardening rules. The model is simulated by a dynamic explicit finite element method, and it is validated with experimental data. For the proposed numerical approach, it analyses different element types, element sizes, and material models. The proposed model is used to simulate the SPIF of three types of polymers (PVC, PC, and High Density Polyethylene HDPE) with different forming capabilities.

The work starts in Section 2, explaining the proposed material and thermal models, and the accomplished simulations. Section 3 includes a description of the experimental setup and the performed tests. The simulated results are in good agreement with experimental results as it is portrayed in Section 4, and the main conclusions are drawn in Section 4.

## 2. Numerical Simulations

The following section describes the numerical procedure, and the numerical simulations run to fit and evaluate the proposed numerical model.

### 2.1. Thermomechanical Model

**Figure 2:**
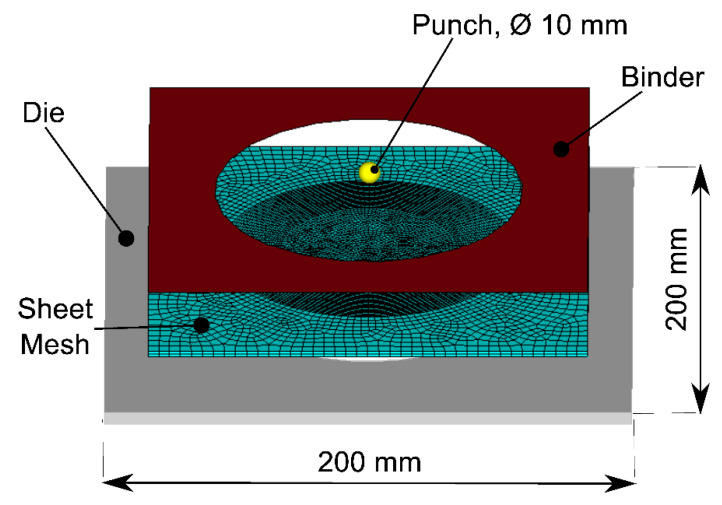
FEM model for the SPIF process.

To obtain a realistic simulation of a SPIF process during polymer sheet forming, we propose a fully coupled thermal-stress model with an explicit integration scheme using the commercial software ABAQUS^®^. It solves the inertia effect, the material temperature-dependent response, and the transient thermal response. During the simulations, the forming tool was defined as an analytical-rigid surface to perform an efficient numerical contact analysis. A penalty contact formulation between the plastic shell and the punch was chosen with a balanced master-slave weighting, for both normal and tangential direction that relates the contact force to the penetration distance. The mesh was designed by considering the tool path to reduce the distortion in elements, employing coupled displacement-temperature elements. The authors selected the smallest element size to limit the computational time to 52 h (for an Intel Core i8 CPU), maintaining an adequate concordance with the experimental results. The characteristic length of the smallest element in the model was 1.25 mm, generating a stable time increment of 2.5 × 10^−7^ s. This element size generates a considerable computational time; for this reason, a fixed mass scaling algorithm with a limitation in the stable time increment of 2.5 × 10^−5^ s was used. Figure 2 shows the FEM model of the SPIF process.

### 2.2. Material Model

A linear elastic model combined with J2-plasticity theory based on isotropic hardening has been used in this work. Please note that according to [17], this model leads an error of 3.8% for semi-crystalline polymer (HDPE) and 7.8% for amorphous polymer (PET) under uniaxial tension test at three different strain rates.

The J_2_-plasticity component is based on isotropic hardening, which describes the size change of the yield surface *σ*^0^ as an exponential function of the equivalent plastic strain ε^pl^:

(1) $$\sigma^{0}=\sigma_{0}+Q_{\infty }\left(1-\exp^{-b\cdot \varepsilon^{\mathrm{pl}}}\right)$$

where *σ*_0_ is the yield stress at zero plastic strain, *Q*_∞_ the maximum change of the size in the yield surface and *b* defines the rate at which the size of the yield surface changes as plastic strain develops.

The implemented model depends on temperature and ε^pl^. It considers that the yield condition is rate-independent, so that *σ*^0^ is equal to von Mises effective stress.

To make affordable and straightforward the implementation of the proposed technique to different materials, the authors use a simple linear hardening rule instead of the exponential law (Equation (1)). This linear law defines the evolution of the yield surface size *σ*^0^ at three different temperatures. Three well-known polymers have been studied so that their material parameters (at different temperatures) can be found easily in the literature (Table 1). Linear fitting of the data listed in Table 1 provides the material parameters (E, σ_y_, σ_ult_, ε_ult_) at a specific desired temperature.

### 2.3. Thermal Simulation

During the forming process, most mechanical work accomplished by the CNC machine used in the SPIF process changes the sheet shape. Still, a percentage is converted into heat by irreversible processes such as friction. The influence of friction during SPIF has been widely discussed in several studies such as [11,22,23]. They concluded that in the SPIF of thermoplastics, friction plays a determinant role due to the increase of material temperature, which reduces the strength and stiffness.

During the numerical simulations performed for this work, we used a constant dynamic coefficient of friction for the unlubricated sheet interface. The stick-slip behavior, which is the results of a hard-contact of metal with a thermoplastic sheet, was not taken into account, due to Park et al. [24] concluded that this effect could be neglected for sliding velocities above 30 mm/min.

The thermal energy released by friction is exchanged among sheet, punch, and the environment. A model is required to define this heat transfer. The thermal contact conductance ψ between the punch (usually a metal forming tool) and the polymer sheet is simulated by gap conductance [25]. We assume natural convection at 20 °C room temperature with a heat transfer coefficient *h* = 0.05 mW/mm^2^ °C. The radiation effect was not taken into account. Other properties such as thermal expansion, thermal conductance *k*_t_, and specific heat *c*_p_, are provided by the polymer datasheet. Table 2 summarizes all thermal properties used in the numerical model.

### 2.4. Numerical Tests

Due to its effect on the numerical model estimations, the influence of element type, element size, and the constitutive material model have been studied. PVC is our reference material in the numerical tests conducted to define the adequate numerical parameters. This thermoplastic is well-known in research works that analyze the resulting forming forces, the predicted deformation mechanism, and temperature distribution in SPIF processes.

Please note that the element type and size have a significant influence on numerical estimations. Different Element types lead to a different force estimation due to the different ability of each element to capture the stress and temperature wake left by the punch through the sheet thickness. On the other hand, it is essential to quantify the element size influences on the accuracy and computational time. For example, Smith et al. [26] reported a simulation of 30 days with aluminum material using an explicit Finite Element Analysis (FEA) scheme and linear bricks elements with a time-step of 1.6 × 10^−6^ s; this great computational time could make the numerical research very tedious.

Table 3 shows the numerical factors tested. The element type tested were three-dimensional 8-nodes linear element (C3D8RT), two-dimensional 4-nodes bilinear element (S4RT), and a three-dimensional 8-nodes linear element, finite membrane strain (SC8RT). All are first-order elements (to reduce the processing time) and have a coupled displacement-temperature formulation with selective reduced integration scheme avoiding the hourglassing. On the other hand, three element’s sizes were tested, the minimum element length of 1.25 mm (small enough to obtain good results, avoiding small step time), 2.5 mm and 5.0 mm. Finally, the proposed model was compared to a complex rheology material for testing the differences in accuracy and computational time.

Additionally, once the numerical model was fitted (regarding the element type-size), it was applied to two different thermoplastic materials, PC and HDPE, for analyzing the performance of the proposed approach.

## 3. Experimental SPIF Tests

The following section describes the experimental procedure and the performed examples.

### 3.1. Experimental Setup

**Figure 3:**
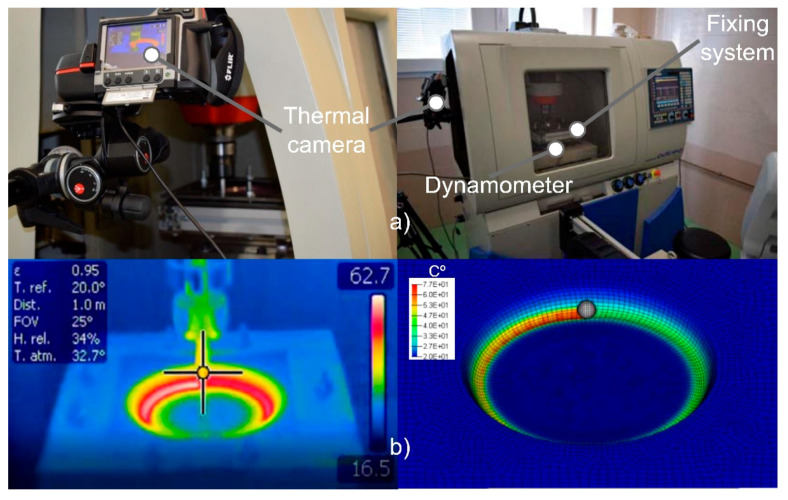
Experimental setup (**a**) and thermal correlation with numerical results (**b**).

SPIF process was accomplished in a conventional CNC milling machine tuned with a SPIF frame and a hemispherical aluminum punch. The milling machine, an ALECOP-ODISEA machine, and the in-house developed fixing system are shown in Figure 3a. The sheet fixing system was placed on the machining bed, and it consists of a frame made of four aluminum profiles, a die with a hole of 140 mm of diameter to obtain truncated cones, and an upper die to fix the sheet. Eight screws were used to fasten the two dies. Regarding the forming tool, it is a 10 mm diameter hemispherical punch made of aluminum 1050-H2.

In the experiments, 3 mm thickness of commercial polymer sheets were formed. The maximum size of the sheet is the same as that of the CNC working space to properly fix the sheet (200 mm × 200 mm). The final shape obtained is a truncated cone *z* = 15 mm of deep, an outer diameter of 130 mm, and a drawing angle α = 60°, below of the maximum angle recommended by Martins et al. [8] for thermoplastic materials. Table 4 summarizes all process parameters of the experimental test.

All experimental tests were conducted without lubricant fluid, and therefore, a high-temperature gradient is localized in the punch-sheet contact. The gradient of temperature localized in the plastic sheet along the wake generated by the steel tip is due to the frictional sliding and the plastic deformation. The temperature increment in the thermoplastic sheet requires a temperature-dependent material model to simulate the softening behavior during forming. Throughout the experimental tests, forces and temperature were measured by a 9257BA Kistler dynamometer table (Kistler Instrumente AG., Winterthur, Swizerland), and a 320 × 240 pixel resolution Flir T335 thermal imaging camera (FLIR^®^ Systems Inc., Wilsonville, OR, USA), respectively (Figure 3).

The resulting shape was captured using a 3D scanner Ein-Scan SP with a single shot accuracy lower than 0.1 mm. From the 3D point cloud, we obtained an internal and external surface and estimated the dimensional deviations to the numerical solution.

### 3.2. Experimental Tests

To fit and validate the developed numerical model, we formed three polymers: PVC (for fitting the model), PC, and High Density Polyethylene HDPE (for validation). Table 4 summarizes the main process parameters for the real tests accomplished. As it is explained above, the incremental forming of the PVC test is also used to choose adequate numerical parameters from those in Table 3.

A programmed MATHEMATICA^®^ function provided the G-code for the SPIF punch-path. At each incremental depth *z* (see scheme in Figure 1), the path consists of a circular arc described by a G2 interpolation. On the other hand, the movement between *z_i_* and *z*_*i*+1_ is a G1 linear interpolation in *x*-*z* from diameter *D_i_* to *D*_*i*+1_.

### 3.3. Error Estimation

The approximation error *e* was estimated as the percentage of the Mean Absolute Error MAE to the mean absolute experimental value *μ*. This metric is also called Weighted Absolute Percentage Error WAPE [27]. Please note that the result of each test was a set of experimental measurements *y_i_* and their corresponding approximations *f_i_* where *i* = {1, 2,…,*m*}, and therefore:

(2) $$e=100\cdot \frac{MAE}{\mu },\;\;MAE=\frac{\sum_{i=1}^{m}\left|y_{i}-f_{i}\right|}{m},\;\mu =\frac{\sum_{i=1}^{m}\left|y_{i}\right|}{m}.$$

## 4. Results and Discussion

### 4.1. Effect of Numerical Model Parameters

#### 4.1.1. Element Type and Size

**Figure 4:**
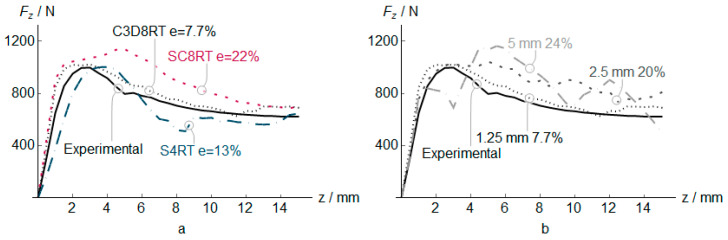
Vertical force *F*_z_ in PVC incremental forming. Experimental vs. predicted mean values at *z* positions, and estimation error *e* for different elements (**a**), and different C3D8RT sizes (**b**).

**Figure 5:**
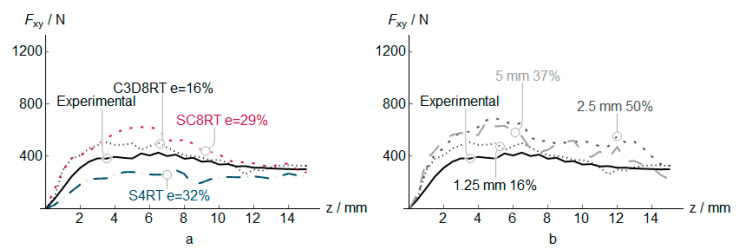
Plane force *F*_xy_ in PVC incremental forming. Experimental vs. predicted mean values at *z* positions, and estimation error *e* for different elements (**a**), and different C3D8RT sizes (**b**).

**Figure 6:**
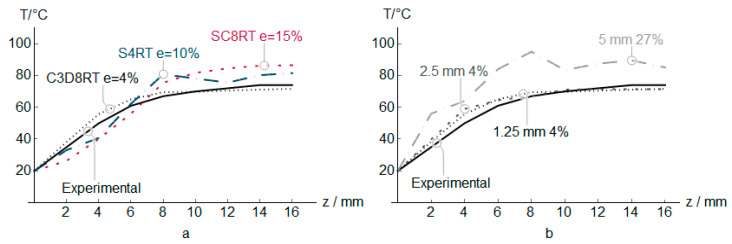
Temperature in PVC incremental forming. Experimental vs. predicted values at *z* positions, and error *e* for different elements (**a**), and different C3D8RT sizes (**b**).

**Figure 7:**
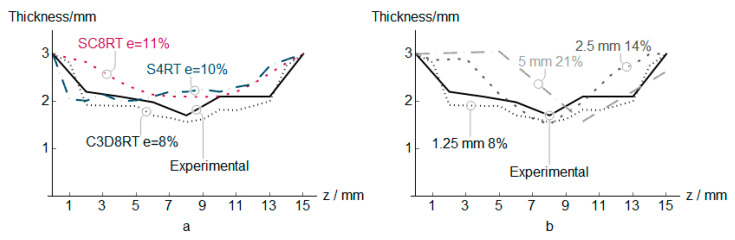
PVC sheet thickness estimation for different element types (**a**) and C3D8RT sizes (**b**).

**Figure 8:**
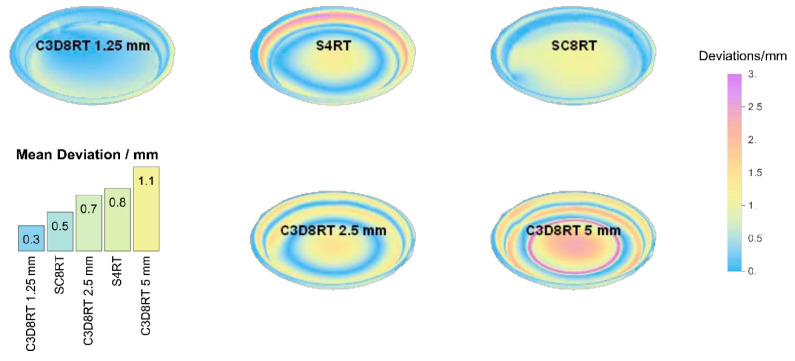
Dimensional deviations measured using 3D scanning for the different element types and sizes.

**Figure 9:**
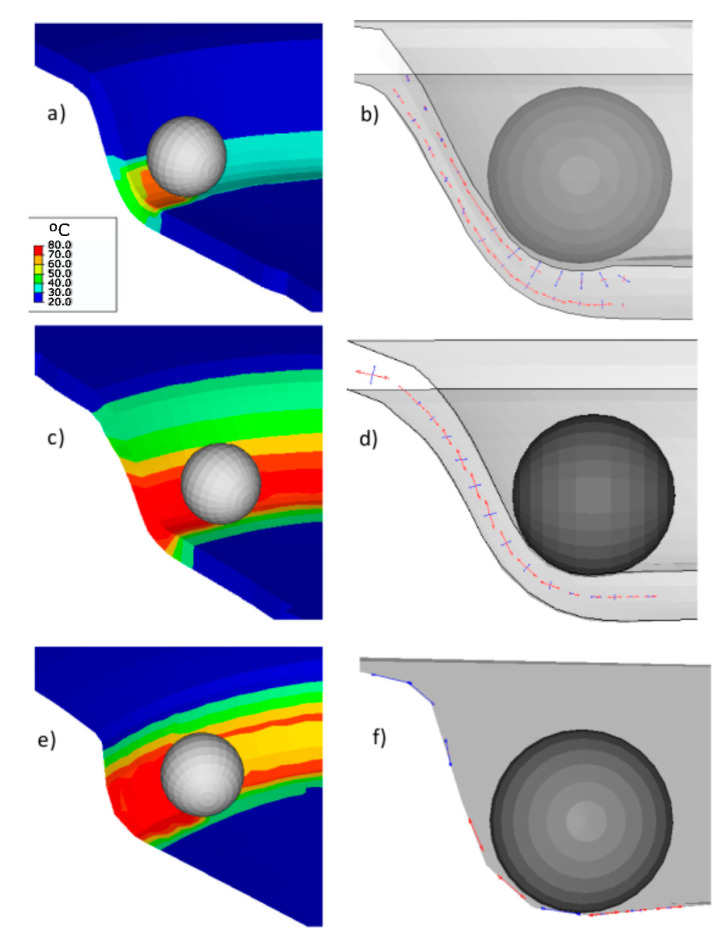
Deformation mechanism. Temperature (left), and strain (right) distribution at *z* = 15 mm for different element types. C3D8RT (**a**,**b**), SC8RT (**c**,**d**) and S4RT (**e**,**f**).

The PVC numerical, along with the experimental measures results (vertical and in-plane forces, temperature, and thickness) for three different element types, are shown in Figure 4, Figure 5, Figure 6 and Figure 7. In addition to that the geometrical deviation between the numerical and experimental shapes can be observed as a 3D color deviation plot in Figure 8. The comparison between numerical and experimental results was carried out in the software MATHEMATICA^®^. Finally, Figure 9 shows some illustrations about the influence of the element type in the deformation mechanism and the temperature distribution.

Regarding the vertical force *F*_z_ (Figure 4), the C3D8RT element provides the best prediction, capturing the softening after the maximum peak located at *z* = 3 mm with an average error of 7.7%. The elements S4RT and SC8RT obtain the peak force at *z* = 4 mm and *z* = 5 mm, respectively, far away from the maximum force obtained by experimental results with an error of 13% and 22% respectively. *F*_z_ reduction from *z* > 3 mm is mainly due to the effect of material softening behavior and the increment of temperature in the material during the forming process (Figure 6). Please note that the force is decreased until the temperature reaches a stable zone. The element length of 1.25 mm shows the most stable response and an excellent correlation with experimental results. Higher element length increases the reaction force response and the instabilities during the analysis generating non-uniform mean reaction force.

The temperature generated by the friction and inelastic deformation is the primary mechanism involved in the reduction of the mean vertical force. Mulliken et al. [28] through a Dynamic Mechanical Analysis (DMA) thermogram of PVC, showed that the ratio between loss and storage modulus (tan δ) increases from 0.015 at room temperatures up to 0.075 at 74 °C that is the maximum temperature registered during the forming process. This increment of temperature is not homogeneous in the whole section (Figure 9), but in the elements in contact with the punch, subjected to compression stress states, exhibits the higher temperature while the elements in the external surface subjected to traction, the increase of temperature is reduced. For this reason, in the range of temperatures studied, the viscoelastic nonlinearities do not have a significant influence on the forming process of PVC.

The in-plane force *F*_xy_ is presented in Figure 5. As in the case of vertical forces, the C3D8RT obtains the best correlation with experimental results. The 1.25 mm elements provide the best estimations as in the *F*_z_ results. Besides, the error is higher than for the *F*_z_ predictions. Please note that we do not evaluate the effects of temperature, pressure, and sliding in the friction coefficient as use a simple Coulomb frictional model, but the introduction of a more advanced friction coefficient could reduce the error in the numerical results [29].

Figure 6 presents the temperature evolution measured in the internal surface in contact with the punch in every vertical step. The temperature generated during the forming grows from *z* = 0 mm to *z* = 12 mm from where it remains more or less stable. The heat dissipation effects reduce the temperature along the wake left by the contact between the punch and the sheet during the forming process. The element C3D8RT shows an excellent correlation with the experimental results (Figure 3b), which is highly important due to the effect of temperature on the strength of the polymers. The shell elements show a weak correlation due to reduced efficiency in the contact formulation between elements and punch. Elements of 1.25 and 2.5 mm generate similar temperature evolution during every vertical step without significant differences with experimental results.

According to Figure 7, the thinning profile of the truncated cone is presented. The thinning distribution for experimental results and elements C3D8RT, SC8RT shows similar characteristics. In this case, C3D8RT overestimates the thinning that was measured as the minimum distance of the 2 element-node projected in the section measured. The solid element C3D8RT shows the best fit along the entire section with an error of 8%, capturing in a good agreement the stable thinning region. An increment of the element size produces a higher error and a different thinning profile, moving the position of the minimum thickness. For the case of 5 mm, the results showing a non-physical result with an increment of thickness, at *z* = 5 mm, higher than the initial sheet thickness.

The color plot in Figure 8 portrays the geometrical deviation between the predicted and the real final shape. The smallest error was obtained by C3D8RT elements with a mean deviation of 0.3 mm. The shell elements and the C3D8RT with an element length of 5 mm show the highest deviation. The increment of the element size produces instabilities during the analysis and leads to a weak correlation between the numerical and the real part.

To understand the deformation mechanism, Figure 9 shows a snapshot of the process at *z* = 15 mm. According to the indicated major (red arrows) and minor (blue arrows) strain located at gauss points, the punch promotes a compression stress state and out of plane shear stress in those elements at the sheet-punch contact. On the other hand, it sets up a stretch in those elements on the external sheet face.

The solid mesh generated with C3D8RT elements captures the compression stress that generates indentation in the contact area between the punch and the sheet. Elements SC8Rt and S4RT can capture the effects of compression under the punch but, cannot capture the effects of indentation (the section under the contact only shows compression instead of compression and stretch).

Regarding the temperature distribution through the sheet thickness (Figure 9, left), the element C3D8RT displays the maximum temperature on the contact side that undergoes compressive stress due to the friction force. It is similar to the experimental results obtained with the thermal camera (Figure 3b). In the case of SC8RT and S4RT, the temperature distribution is not well defined, showing similar temperatures in the external and internal sides.

#### 4.1.2. Effect of Material Model

The material model plays an important role in the prediction of the response during the forming process. Several factors guide the choice of the best material model for SPIF applications, such as material data available for model calibration (tension test, compression, creep, relaxation, and so on), the computational cost of the model, and the accuracy.

During recent years, several viscoplastic material models were developed for glassy and amorphous polymers [15,30]. These material models exhibit a high accuracy for a wide range of polymers but are numerically expensive and require more experimental data for a satisfactory calibration.

**Figure 10:**
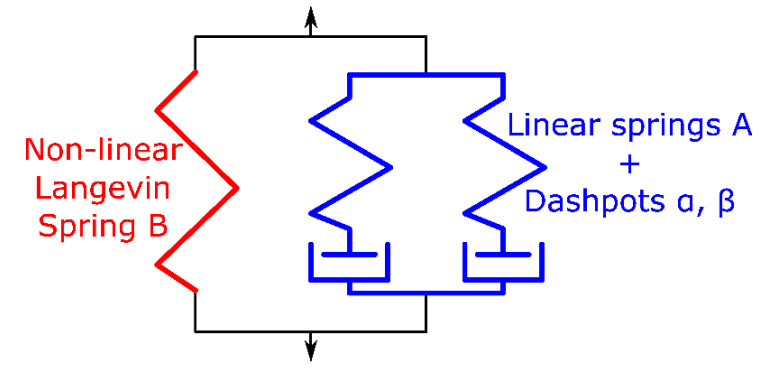
One-dimensional rheological constitutive model [20].

The results obtained for PVC forming with the proposed elastoplastic material with a J-2 isotropic hardening plasticity model were compared against those provided by the Mulliken and Boyce [20] MB model. This model consists of two networks (Figure 10): two linear elastic springs with dashpots in series, and a non-linear Langevin spring. Mulliken and Boyce [20] work explains how to include a temperature rate term and provides the material parameters’ values for PVC. The`MB model is a full three-dimensional model implemented by a vumat user subroutine in ABAQUS using Mcalibration^®^ software from veryst engineering. 

The stress, $\sigma_{j}$ (j=α,β), in the elastic spring are denoted by:

(3) $$\sigma_{j}=E_{j}\varepsilon^{e},$$

where $\varepsilon^{e}$ is the total strain, $E_{i}$ is the temperature-rate dependent Young’s modulus. The viscoplastic behavior in the network (A) is prescribed by two constitutive laws. Relating the shear stress, $\tau_{i}$, with the shear strain rate, ${\dot{\gamma }}_{i}^{p}$:

(4) $${\dot{\gamma }}_{j}^{p}={\dot{\gamma }}_{0,j}^{p}exp\left[-\frac{\Delta G_{j}}{kT}\left(1-\frac{\tau_{j}}{s_{j}+\alpha_{p,j}p}\right)\right],$$

where $\dot{\gamma_{0,j}^{p}}$ is the pre-exponential factor proportional to the attempt frequency; $\Delta G_{j}$ is the activation energy; *k* is the Boltzmann’s constant, *T* is the temperature, *p* is the pressure and $\alpha_{p,j}$ is the pressure coefficient. The internal variable, $s_{j}$, is the athermal shear strength, related to the shear modulus, $\mu_{j}$, and evolves toward a preferred state with plastic straining according to:

(5) $${\dot{S}}_{j}=h_{j}\left(1-\frac{S_{j}}{S_{ss,j}}\right){\dot{\gamma }}_{j}^{p},$$

The initial value of, $s_{j}$ that simulates strain softening is given by:

(6) $$s_{0,j}\equiv \frac{0.077\mu_{j}}{1-\nu_{j}},$$

where $h_{j}$ is the softening slope, $S_{ss,j}$ is the “preferred state”, and $\nu_{j}$ the Poisson´s ratio. The elastic and viscoplastic strain are combined for each component, α, and β and rearranging with (3) to obtain the total stress of the network (A):

(7) $${\dot{\sigma }}_{j}=E_{j}\left(\dot{\varepsilon }-{\dot{\varepsilon }}_{j}^{p}\right).$$

Equations (3) and (7) are solved simultaneously as a system of time-dependent differential equations to determine the stress in the α and β components. The stress response in the non-linear Langevin spring, $\sigma_{B}$, is defined using Arruda & Boyce 8-chain model [30] for the interpretation of molecular alignment:

(8) $$\sigma_{B}=\frac{C_{R}}{3}\frac{\sqrt{N}}{\lambda_{chain}^{p}}L^{-1}\left(\frac{\lambda_{chain}^{p}}{\sqrt{N}}\right){\bar{\overset{´}{B}}}_{B},$$

where $\lambda_{chain}^{p}=\sqrt{trace\left({\bar{B}}_{B}\right)/3}$ is the stretch on a chain in the eight-chain networks; L is the Langevin function defined by $L\left(\beta \right)\equiv \coth\beta -\frac{1}{\beta };$ ${\bar{\overset{´}{B}}}_{B}$ is the deviatoric part of the isochoric left Cauchy-Green tensor, ${\bar{B}}_{B}={\left(detF\right)}^{-2/3}FF^{T};$ $\sqrt{N}$ is the limiting chain extensibility; and $C_{R}\equiv nkT$ is the rubbery modulus (where *n* is the number of chains per unit volume). The total stress in the polymer is given as:

(9) $$\sigma =\sigma_{B}+\sigma_{\alpha }+\sigma_{\beta }.$$

To take into account the increase of temperature, [20] added a temperature rate term to the model:

(10) $$\dot{\sigma }=\frac{1}{\rho c_{p}}\left[\left({\dot{\tau }}_{\alpha }\gamma_{\alpha }^{p}+\tau_{\alpha }{\dot{\gamma }}_{\alpha }^{p}\right)+\left({\dot{\tau }}_{\beta }\gamma_{\beta }^{p}+\tau_{\beta }{\dot{\gamma }}_{\beta }^{p}\right)\right].$$

The model parameters used to capture the material behavior of PVC are given in Table 5, as defined [28].

**Figure 11:**
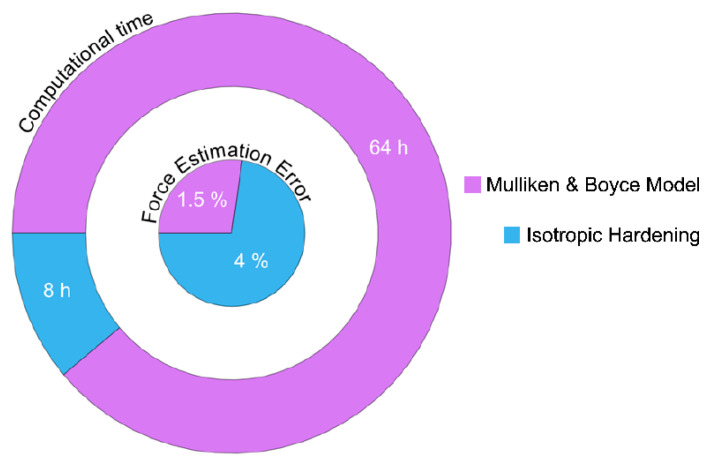
Mulliken and Boyce [20] material vs. the proposed elastoplastic isotropic hardening approach in PVC sheets regarding computational time and maximum *F*_z_ estimation error.

The considerable computational time (64 h) required by the rheological model to reach *z* = 3 mm, where the maximum reaction force is obtained, makes it impossible to complete the simulation. Figure 11 reports the computational time and the *F*_z_ estimation error of the MB and the Isotropic hardening models. The maximum reaction force obtained experimentally was 997 N, the MB model produces 1020 N and the isotropic hardening 975 N, in both cases, the error was less than 5%. As discussed for the Element type and size tests, the effect of the element length in the computational time has a decisive role in the computational time. Element length of 2.5 and 5 mm reduces the simulation time in 6.20 and 2.10 h, respectively, at the expense of a higher error.

**Figure 12:**
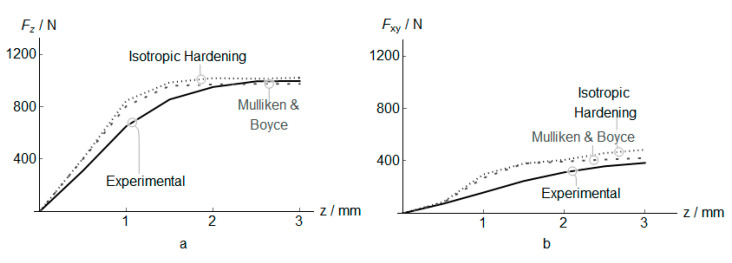
Mean force during PVC incremental forming at *z* positions. Vertical force (**a**), and in-plane force (**b**).

Figure 12a displays the *F*_z_ predicted by the classical plasticity constitutive model with isotropic hardening (kinematic hardening produces a similar mean force, and it is not presented) and by the MB model. The main differences can be noted along the first two millimeters of forming. The MB model shows a lower mean reaction force, due to the ability of the material model to capture stress relaxation and fluence effects in the thermoplastic at low temperatures, obtaining a better correlation with experimental results. The Isotropic hardening neglected these effects during the entire process with has less influence in the force as the temperature rises. Similar results are observed for the in-plane force (Figure 12b).

**Figure 13:**
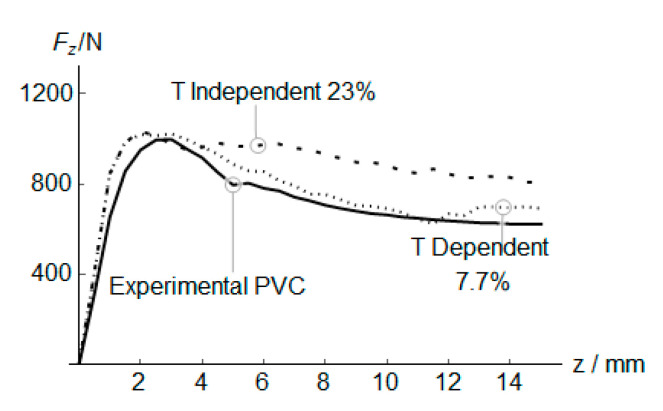
Experimental vs. predicted mean *F*_z_ values and estimation error *e* of temperature-dependent and temperature-independent models for PVC.

Finally, the reaction force both in temperature-dependent and temperature-independent material models have been compared (Figure 13). It is worth mentioning the effect of the coupled temperature-displacement model on the vertical reaction force *F*_z_. The simulation was performed using 1.25 mm C3D8RT elements.

**Figure 14:**
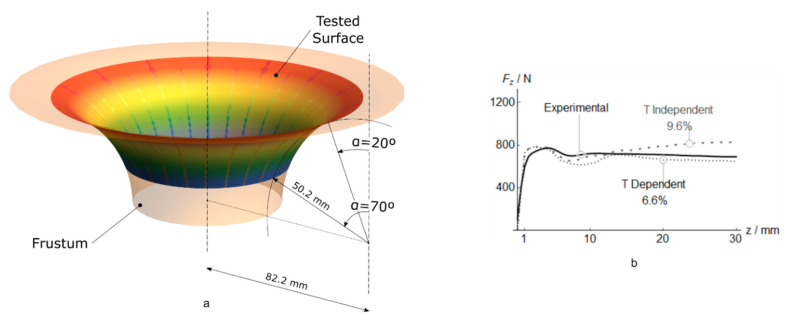
Representation of the Frustum (**a**), Experimental vs. predicted mean *F*_z_ values and estimation error *e* of temperature-dependent and temperature-independent models for PVC during Frustum forming (**b**).

Both materials show a similar reaction force up to *z* = 2.5 mm due to the reduced temperature registered on the sheet (40 °C). The growth of punch-sheet friction and plastic work with z increase the temperature. The temperature increase and the strain-softening material behavior generate in the temperature-dependent material a softening effect of the vertical reaction force, which obtains an error of 7.7% respect to the experimental observation. This error is one-third of the error obtained with a temperature-independent material. To evaluate the performance of the model using different wall angles and the contact surface between the punch and the blank sheet, a Frustum (Figure 14a) where performed. The experimental parameters: punch diameter, incremental depth and feed rate speed were similar to those described in Table 4. The mean *F*_z_ values using temperature-dependent and independent material is showed in Figure 14b. The best correlation was obtained at high wall angles, where the contact surface and friction coefficient is similar to the cone validated previously. 

### 4.2. Validation Tests

Additional tests with two different polymers, commonly used in SPIF applications, were accomplished to validate the numerical procedure. The first polymer is a semi-crystalline thermoplastic HDPE with low strength and high thermal conductance that exhibits a low reaction force and excellent formability. The second material is an amorphous thermoplastic PC with high strength and low thermal conductance, showing a similar reaction force than PVC.

**Figure 15:**
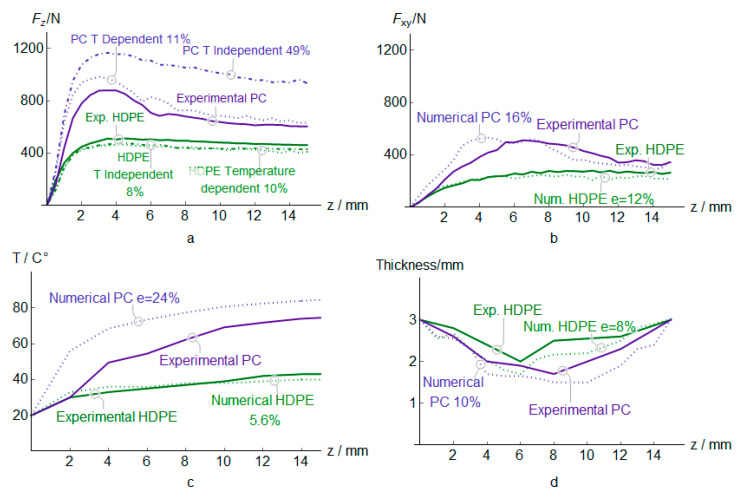
Force *F*_z_, *F*_xy_ (**a**,**b**), temperature (**c**), and thickness (**d**) estimations for PC and HDPE sheets.

Regarding the experimental curves depicted in Figure 15, HDPE show the best formability with a vertical force well-bellow to PC and PVC, and an in-plane force below PC and PVC. The temperature is also lower for the incremental forming of HDPE, while PC has a similar maximum temperature than PVC. Hence, HDPE exhibits lower temperature-dependence during the forming process. Finally, HDPE shows the lowest thinning (Figure 15d), and PC and PVC have a similar minimum value.

Force predictions for HDPE produce an error of 10 % and 12% for *F*_z_ and *F*_xy_ reaction force respectively while PC obtains 11% and 16% (Figure 15a,b). The lower stiffness of HDPE sheets generates low *F*_xy_ forces by the friction punch. This material exhibits a reduced temperature during the forming process, in this case, the softening of the mean reaction force after the peak is negligible and the force remains more or less stable during the process. The *F*_z_ of PC undergoes higher relaxation compared with HDPE due to the higher strength and stiffness of the material, which increases the temperature generated during the process. Regarding the *F*_xy_ force, the HDPE and PC revealed a better correlation with experimental results.

Figure 15a also shows that a temperature-dependent material it is essential for PC due to it has a low thermal conductance and high strength. On the other hand, the use of a temperature-dependent or independent material has a weak influence in HDPE because it has high thermal conductance and low strength. These facts also explain the prediction errors noted in the forces and temperature, which always are higher for PC than for HDPE. Finally, the evolution of the thinning reports similar error than in the case of PVC for both materials, Figure 15d.

## 5. Conclusions

The simulation of the SPIF process for polymers is possible with a thermo-mechanical numerical model as proposed by this work. The results evidence the ability of classical isotropic hardening models to predict reaction forces, temperature, and final shapes. These models are less computationally demanding than complex rheological models.

It is worth mentioning the discussion of the influence of different element types, element size, material models, and the testing of three polymers: PVC (reference material), HDPE, and PC (for model validation). The description of the mechanical and thermal parameters needed in the simulation and the approach followed in this work to determine their values can help other researchers interested in numerical simulation.

The following bullets summarize the main conclusions:

- This model is an improvement in the analysis of SPIF with glassy and amorphous polymers using FEM. It is possible to simulate complex geometries with a reduced error avoiding rheological material models that require a large number of material parameters. The proposed model obtains a reduced error (*e* < 11% for vertical forces) with a viable computational time (8 h up to *z* = 3 mm).
- The element type plays a highlighted role in the prediction of material behavior. 3D solid elements capture the indentation effects in the contact area between the punch and the sheet, the increase of temperatures generated, and the final shape with excellent agreement between numerical and experimental results.
- The element length should be taken into consideration. The computational time is an important issue that needs to be evaluated. Larges elements reduce the computational time dramatically, generating a higher error in the reaction forces and final shapes prediction.
- The results point to the likelihood that the method proposed can be applied to all glassy and amorphous polymers in SPIF applications, considering the results presented using PVC and PC thermoplastic. The evidence from this study suggests the application of temperature-dependence material in the simulation of SPIF of the abovementioned polymers with considerable thermal conductance.
- The method can simulate all thermoplastics, but low strength materials such as HDPE undergoes low *F*_xy_ forces, developing low material temperature during the forming process that reduces the influence of the proposed method.

## Figures and Tables

**Table 1:** Mechanical properties of PVC, PC, and HDPE at different temperatures.

Material	Temperature (°C)	Young Modulus E (MPa)	Yield Stress σ_y_ (MPa)	Ultimate Strength σ_ult_ (MPa)	Ultimate Strain ε_ult_
PVC [18]	Room Temp.	2775	64	38	0.17
	40	2109	57	33	0.14
	70	1592	31	12	0.4
PC [19,20]	Room Temp.	2351	68	70	0.3
	80	2072	52	60	0.4
HDPE [21]	Room Temp.	515	26	23	0.5
	30	445	24	21	0.5
	50	360	19.5	19	0.5

**Table 2:** Thermal parameters used in the model and friction coefficient.

Properties	HDPE	PC	PVC
Gap Conductance (W/m^2^·K)	30 × 10^−6^	30 × 10^−6^	30 × 10^−6^
Thermal conductance (W/m·K)	0.44	0.2	0.175
Specific heat (kJ/kg K)	1.33	1.25	1.18
Thermal expansion (1/K)	12 × 10^−5^	6.2 × 10^−5^	7 × 10^−5^
Friction coefficient	0.15	0.33	0.2

**Table 3:** Element type, element size, and material model tested in the numerical tests.

Tested Factors		
Element Type	Element Size (of the Best Element Type)	Material Model
C3D8RT S4RT SC8RT	1.25 mm 2.5 mm 5.0 mm	Mulliken& Boyce Isotropic Hardening

**Table 4:** Process parameters used in the experimental tests.

Experimental Test Parameters	Value
Cone wall angle, α	60°
Punch diameter, *D*	10 mm
Incremental depth, *z*	0.5 mm
Total depth	15 mm
Feed rate speed	4500 mm/min
Cone initial diameter	140 mm

**Table 5:** Initial parameters for the MB model applied to PVC [28].

Material Parameters			
$E_{\alpha }$($\dot{\varepsilon },\theta )$	DMA data	$\Delta G_{\alpha }\left[J\right]$	3.1 × 10^−19^
$E_{\beta }$($\dot{\varepsilon },\theta )$	DMA data	$\Delta G_{\beta }\left[J\right]$	9.19 × 10^−20^
$v_{\alpha }=v_{\beta }$	0.38	$h_{\alpha }\left[\mathrm{MPa}\right]$	450
$\alpha_{p,\alpha }=\alpha_{p,\beta }$	0.22	$s_{ss}/s_{0}$	0.53
${\dot{\gamma }}_{0}^{\alpha }\left[s^{-1}\right]$	1.0 × 10^−19^	$\sqrt{N}$	2.9
${\dot{\gamma }}_{0}^{\beta }\left[s^{-1}\right]$	8.2e6	$C_{R}\left[\mathrm{MPa}\right]$	13.0
$c_{p}\left[J/\mathrm{kg}\cdot K\right]$	2200		

## Nomenclature

*b*	Change rate of *σ*^0^
${\bar{\overset{´}{B}}}_{B}$	Deviatoric part of the isochoric left Cauchy-Green tensor (MPa)
*c*_p_	Specific heat (kJ/kg·K)
$C_{R}$	Rubbery modulus (MPa)
*D*	Punch diameter (mm)
*e*	Approximation error
*f*_i_	Predictions
*F*_z_	Vertical force (N)
*F*_xy_	In-plane force (N)
$h_{j}$	Softening slope (MPa)
*k*_t_	Thermal conductance (W/m·K)
L	Langevin function
*n*	Chain density (polymer chains per unit volume)
$\sqrt{N}$	Limiting chain extensibility
*p*	Pressure (MPa)
*Q*_∞_	Maximum *σ*^0^ (MPa)
$s_{j}$	Athermal shear strength (MPa)
*T*	Temperature (°C)
*y*_i_	Experimental measurements
*z*	Incremental depth (mm)
Greek symbols	
α	Cone wall angle (°)
$\alpha_{p,j}$	Pressure coefficient
${\dot{\gamma }}_{i}^{p}$	Shear strain rate in the viscoplastic dashpot (1/s)
${\dot{\gamma }}_{0,j}^{p}$	Pre-exponential factor proportional to the attempt frequency (1/s)
$\Delta G_{j}$	Activation energy (J)
ε^e^	Total strain
ε^pl^	Equivalent plastic strain
ε_ult_	Ultimate strain
E	Young’s modulus (MPa)
E_j_	Temperature rate dependent Young’s modulus (MPa)
*k*	Boltzmann’s constant (J/K)
$\lambda_{chain}^{p}$	Stretch on a chain in the eight-chain networks (MPa)
μ	Mean absolute experimental value
$\mu_{j}$	Shear modulus (MPa)
$\nu_{j}$	Poisson´s ratio
σ	Total stress in the polymer (MPa)
$\sigma_{B}$	Stress response in the non-linear Langevin spring (MPa)
$\sigma_{j}$	Stress in the linear elastic spring (MPa)
σ^0^	Size change of the yield surface (MPa)
σ_0_	Yield stress at zero plastic strain (MPa)
σ_ult_	Ultimate strength (MPa)
σ_y_	Yield stress (MPa)
$\tau_{j}$	Shear stress (MPa)
ψ	Thermal contact conductance (W/m^2^·K)

## Abbreviations

FFLD	Fracture Forming Limit Diagrams
HDPE	High Density Polyethylene
ISF	Incremental Sheet Forming
MAE	Mean Absolute Error
MB	Rheological model proposed by Mulliken and Boyce (2006)
PA	Polyamide
PC	Polycarbonate
POM	Polyoxymethylene
PVC	Polyvinyl Chloride
SPIF	Single Point Incremental Forming
WAPE	Weighted Absolute Percentage Error
